# Neuromodulation in new-onset refractory status epilepticus

**DOI:** 10.3389/fneur.2023.1195844

**Published:** 2023-06-14

**Authors:** Ioannis Stavropoulos, Jin Han Khaw, Antonio Valentin

**Affiliations:** ^1^Department of Basic and Clinical Neuroscience, Institute of Psychiatry, Psychology and Neuroscience, King's College London, London, United Kingdom; ^2^Department of Clinical Neurophysiology, King's College Hospital, London, United Kingdom; ^3^Faculty of Life Sciences and Medicine, King's College London, London, United Kingdom

**Keywords:** new-onset refractory status epilepticus (NORSE), febrile infection epilepsy-related syndrome (FIRES), neuromodulation, deep brain stimulation (DBS), electroconvulsive therapy (ECT), vagal nerve stimulation (VNS)

## Abstract

**Background:**

New-onset refractory status epilepticus (NORSE) and its subset of febrile infection-related epilepsy syndrome (FIRES) are devastating clinical presentations with high rates of mortality and morbidity. The recently published consensus on the treatment of these conditions includes anesthetics, antiseizure drugs, antivirals, antibiotics, and immune therapies. Despite the internationally accepted treatment, the outcome remains poor for a significant percentage of patients.

**Methods:**

We conducted a systematic review of the use of neuromodulation techniques in the treatment of the acute phase of NORSE/FIRES using the Preferred Reporting Items for Systematic Reviews and Meta-Analyses (PRISMA) guidelines.

**Results:**

Our search strategy brought up 74 articles of which 15 met our inclusion criteria. A total of 20 patients were treated with neuromodulation. Thirteen cases represented FIRES and in 17 cases the NORSE remained cryptogenic. Ten had electroconvulsive therapy (ECT), seven had vagal nerve stimulation (VNS), and four had deep brain stimulation (DBS); one patient had initially VNS and later DBS. Eight patients were female and nine were children. In 17 out of 20 patients, the status epilepticus was resolved after neuromodulation, while three patients died.

**Conclusion:**

NORSE can have a catastrophic course and the first treatment goal should be the fastest possible termination of status epilepticus. The data presented are limited by the small number of published cases and the variability of neuromodulation protocols used. However, they show some potential clinical benefits of early neuromodulation therapy, suggesting that these techniques could be considered within the course of FIRES/NORSE.

## Introduction

The Neurocritical Care Society has described status epilepticus (SE) as one of the most frequent neurological emergencies defined as a seizure with 5 min or more of continuous clinical and/or electrographic seizure activity or recurrent seizure activity without recovery between seizures (1). SE is a condition resulting either from the failure of the mechanisms responsible for seizure termination or from the initiation of mechanisms, which leads to prolonged seizures (2). SE has an incidence ranging between 8.52 and 41/100,000/year according to a recent review (3). This significant discrepancy between the different studies could be attributed to different study methodologies, populations, geographical representation, and also different SE definitions. A study in adults using the latest definition for SE from the International League Against Epilepsy (ILAE) found an incidence of 36.1/100,000 adults per year (4).

The mortality incidence of this condition increases dramatically when it persists and becomes refractory (RSE) or super refractory SE (SRSE). RSE is defined as the persistence of SE after the administration of two parenteral medications including a benzodiazepine and its termination requires general anesthesia (2, 5). SRSE is defined as the persistence of SE for 24 hours after administration of anesthesia, which could be uninterrupted or “recurring while on or after withdrawal of anesthesia, requiring anesthetic reintroduction” (5, 6). About 20% of the RSE cases will evolve to SRSE (7), which has a mortality rate of 30–50% in different studies (6, 8), and thus a rapid diagnostic assessment and appropriate treatment are of major importance for the best possible outcome.

New-onset refractory status epilepticus (NORSE) is defined as a “clinical presentation, not a specific diagnosis, characterized by de novo onset of RSE that may progress toward SRSE, in a patient without active epilepsy or other pre-existing relevant neurological disorders, and without an identifiable acute or active structural, toxic, or metabolic cause” (5). In the same article, febrile infection-related epilepsy syndrome (FIRES) has been defined as a subset of NORSE “requiring a febrile illness starting between 2 weeks and 24 h before the onset of RSE, with or without fever at the onset of SE” (5). There are no age restrictions to both NORSE and FIRES. Historically, several syndromes have also been used to describe similar cases of fever preceding RSE, such as de novo cryptogenic refractory multifocal febrile status epilepticus (9), idiopathic catastrophic epileptic encephalopathy (10), severe refractory status epilepticus owing to presumed encephalitis (11), devastating epilepsy in school-age children (12), acute non-herpetic encephalitis with refractory repetitive seizures (13), acute encephalopathy with inflammation-mediated status epilepticus (14), and acute encephalitis with refractory repetitive partial seizures (15). In a review of 249 cases named under these nomenclatures, Ismail and Kossoff (16) concluded that they represent the same clinical entity of FIRES.

NORSE remains without an identifiable cause in 50–73% of the cases and typically is called cryptogenic NORSE (17–19). It is a devastating condition with a mortality rate between 10 and 30% and about two-thirds of the survivors will have functional and cognitive impairment (20). Epilepsy persists after SE resolution in about 80% of the patients (18). In a retrospective review of 130 patients with NORSE, 22% of affected patients died in the hospital, and 62% had a poor outcome on discharge (19). Cryptogenic NORSE has even poorer outcomes (18). Various treatment options have been described in the literature apart from the common SE treatment with benzodiazepines, antiseizure drugs, and anesthetics. These include immune therapies such as methylprednisolone, therapeutic plasma exchange (TPE), and intravenous immunoglobulin (IVIG); hypothermia, ketogenic diet, second-line immunomodulatory treatments (anakinra, rituximab), surgical resection, and neuromodulation. However, no standardized approach existed until the international consensus recommendations for the management of NORSE/FIRES that were published recently (21). The treatment suggestions and their timeline are described in detail (22). Besides the antiseizure medications, the anesthetics, and the management of possible infection, there is a suggestion for initiation of first-line immunotherapy (corticosteroids or IVIG) within 72 h if basic infections have been excluded. Ketogenic diet and second-line immunotherapies should be initiated within 7 days of NORSE/FIRES onset. The guidelines do not include any neuromodulation technique during the acute phase of NORSE/FIRES based on the existence of limited data (21, 22). Although the authors suggest vagal nerve stimulation (VNS) for the post-acute phase, they do not suggest deep brain stimulation (DBS). Nevertheless, it is stated that there is no evidence of lack of efficacy for the latter (21, 22).

The need for complementary non-pharmacological treatments has been described in general for SRSE (23) and applies with higher importance to NORSE/FIRES as they can have potentially catastrophic consequences for the patient. There is a small number of published cases where neuromodulation was used for the treatment of NORSE/FIRES. Both non-invasive (electroconvulsive therapy [ECT]) and invasive techniques (VNS and DBS) have been applied. Other non-invasive techniques such as transcranial magnetic stimulation (TMS), transcranial direct electrical stimulation (tDCS), and external VNS have not been reported for NORSE/FIRES.

### Non-invasive neuromodulation techniques

Electroconvulsive therapy (ECT) was primarily used in the past to treat patients with severe major depression, schizophrenia, catatonia, and many other mental disorders with high efficacy (24–28), but recent reviews have demonstrated good outcomes when used to abolish RSE or SRSE (29, 30). This non-invasive technique involves transcutaneous electrical stimulation of the cerebral cortex to induce a generalized seizure under EEG monitoring with general anesthesia. The ECT stimulus intensity and duration (pulse width) are determined by the patient's seizure threshold through trial and error, which affects efficacy, response speed, and severity of adverse cognitive effects (31). There are three types of electrode placement: bifrontal, bitemporal, and right unilateral (left unilateral for left-handed patients). Bitemporal placement is preferred in urgent clinical situations due to its higher speed of response, while right unilateral placement in situations where minimizing retrograde amnesia is a concern (27). The aim is to increase the patient's seizure threshold, potentially by 80% with bilateral ECT or 40% with unilateral ECT over one treatment course (32).

### Invasive neuromodulation techniques

Vagal nerve stimulation (VNS) is an add-on treatment approved by the National Institute for Health and Care Excellence (NICE) for children and adults suffering from drug-resistant epilepsy (33). A pulse generator with a battery is implanted in the left subclavicular area and is connected with a 43-cm lead wire to two platinum/iridium helical electrodes attached to the left vagus nerve. An external programming system is used to control stimulation parameters (34). The reported early complications include bradycardia/asystole during the implantation procedure, peritracheal hematoma, and infections (3–8%) (34).

Deep brain stimulation (DBS) is an invasive neuromodulation technique approved by the United States Food and Drug Administration (FDA) for treating movement disorders (such as Parkinson's disease, essential tremor, and dystonia), treatment-refractory obsessive-compulsive disorder, chronic pain, and epilepsy (35–37). NICE has approved only anterior thalamic nucleus DBS for the treatment of refractory epilepsy in adults when pharmacological options have failed and resective surgery is contraindicated (38). The apparatus consists of electrodes with multiple contacts implanted to specifically targeted deep brain structures and connected through a subcutaneous wire to a pulse generator implanted on the chest wall. Stimulation parameters consisting of electrical (voltage or constant current) pulses with different amplitudes, frequencies, and pulse widths are controlled by an external wireless device (39). In patients with drug-resistant epilepsy, RSE, or SRSE, the electrodes are commonly implanted at the anterior or centromedian thalamic nucleus (37, 40). Possible hardware-related complications include lead migration or fracture, internal pulse generator malfunction, and skin erosion. As stated in a recent review (41), the most frequent complication is infection related to the implantation (≈5%).

This systematic review aims to present the available data about the possible benefits of using neuromodulation techniques as an add-on non-pharmacological treatment in cases of NORSE/FIRES.

## Methods

**Figure 1:**
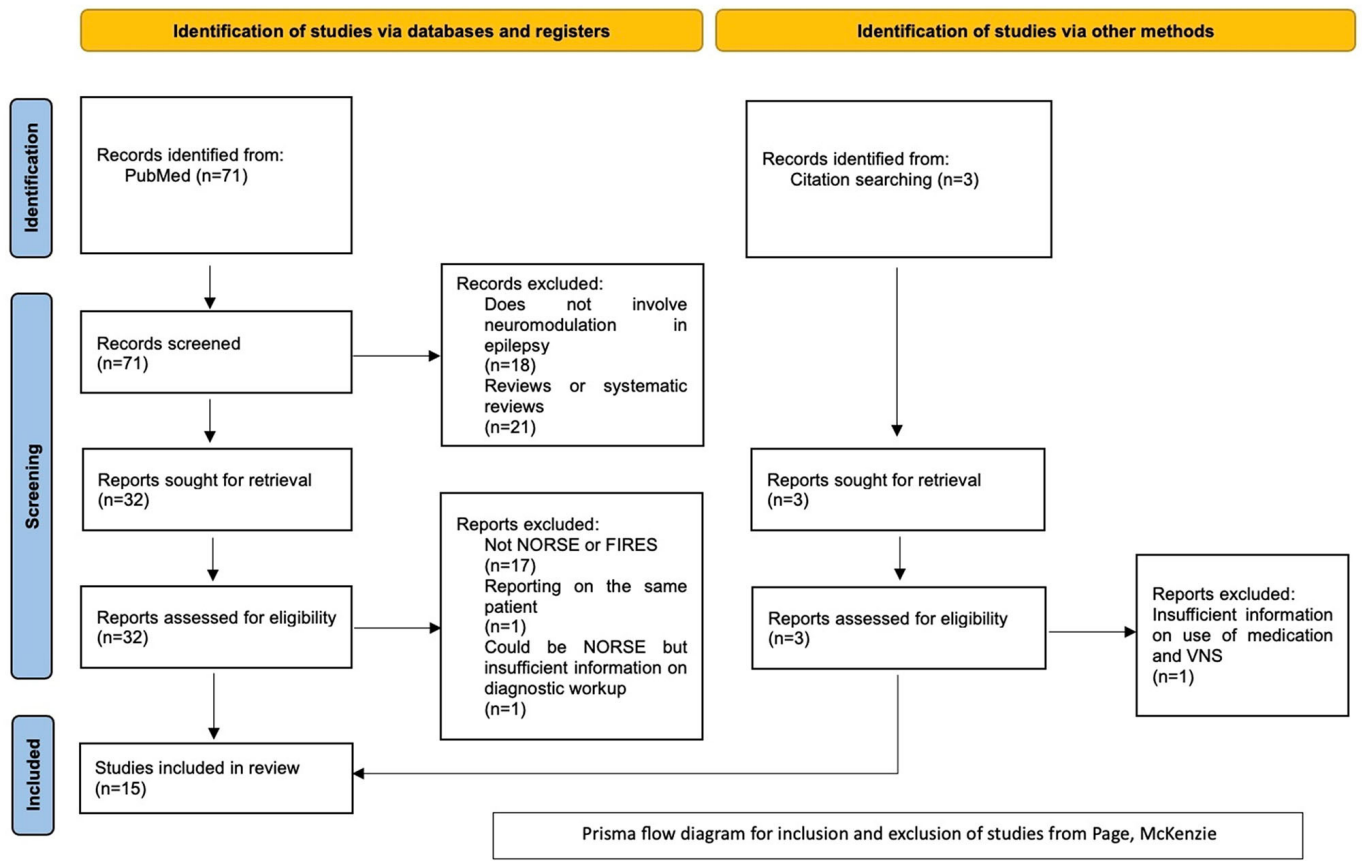
PRISMA flowchart for choice of included studies.

This systematic review was performed in line with the Preferred Reporting Items for Systematic Reviews and Meta-Analyses (PRISMA) guidelines (42). The inclusion criteria were full-length articles written in English. These could be original articles, letters to the editor, or case reports/series. Articles containing overlapping data from previously published original articles, conference abstracts, and review articles were excluded. The studied population is patients with NORSE or FIRES who had treatment with any neuromodulation technique during the acute phase, defined as being still in SE, in the intensive care unit (ICU), and under sedation. NORSE cases with fever at the admission but without declaring when the fever started were considered as NORSE and not as FIRES because it was unclear whether the fever had started at least 24 h before the onset of RSE. Moreover, we have included cases where the authors did not use the terms NORSE or FIRES, but they have described the clinical details and testing approach and the condition could fit the current NORSE/FIRES definition. Articles without basic information about diagnostic or treatment approaches were excluded (Figure 1). Good outcome was considered the termination of SE irrelevant to the final outcome for the patient.

The search strategy is described in detail in Appendix 1.

## Results

This review includes data from a total of 20 patients, but one patient underwent both VNS and DBS (43). Therefore, there were 21 neuromodulation treatments administered across 15 studies. The neuromodulation techniques used were ECT (*n* = 10), VNS (*n* = 7), and DBS (*n* = 4). Twelve out of 20 patients were male. Nine out of 20 patients were children (< 18) and all except two (44, 45) had a diagnosis of FIRES. Seven out of 20 patients presented with NORSE, and the etiology was confirmed only in three (45–47) out of 20, with all the other cases remaining cryptogenic. A complete breakdown of demographics and outcomes is illustrated in Table 1. In cases where the patient experienced a good outcome, the time from neuromodulation until the improvement was reported heterogeneously. Some studies reported time before weaning anesthetics without any clinical or electrical seizure recurrence, some others reported the time before the discharge of the patient from neurointensive care, while others reported when SE was resolved.

**Table 1:** Basic demographics and outcomes.

**Age, years, mean (range)**	**ECT**	**28.6 (3–77)**
	VNS	22.9 (3–46)
	DBS	10.5 (5–17)
	**Entire cohort**	**23.8 (3**–**77)**
Neuromodulations (*n* = number of patients)	ECT	10
	VNS	7
	DBS	4
Gender (*n* = number of patients)	Males	12
	Females	8
NORSE or FIRES (*n* = number of patients)	NORSE	7
	FIRES	13
Etiology (*n* = number of patients)	Known (Encephalitis, CVID^*^)	3
	Cryptogenic	17
	**Total**	**20**
Clinical and/or EEG improvement after neuromodulation (*n* = number of neuromodulation treatments)	Yes	18
	No	3
	**Total**	**21**
Median number of days from NORSE onset to the initiation of neuromodulation (*N* = 20; range 5–435)	ECT	30
	VNS	22^**^
	DBS	47
	**Entire cohort**	**30**
Median number of days from initiation of neuromodulation to SE resolution (*N* = 17; range 0–61)	ECT	8.5
	VNS	7^**^
	DBS	8
	**Entire cohort**	**7**

* Common variable immunodeficiency-associated encephalomyelitis.

** One case patient had VNS very late in the course of NORSE (day 435) (47). When this outlier is excluded, the median number of days from NORSE onset until the treatment with VNS was 14, and from initiation of VNS treatment until SE termination was 5.

The mean duration from NORSE onset to the application of neuromodulation was 56 days with a median of 30 days. Eighteen out of the 20 patients had improvement after neuromodulation [21 treatments, as one patient had VNS which failed, followed by successful DBS (43)], defined as resolution of SE and/or being able to step down from ICU. One of the patients with a resolution of SE after neuromodulation died from other comorbidities (48). The mean time from initiation of neuromodulation until SE resolution was 14 days and the median was seven days (for timings related to each neuromodulation technique see Table 1).

Regarding the overall outcomes, of the 17 survivors, 11 had persistent epilepsy (43–45, 49–54), 12 had cognitive and/or motor dysfunction (43, 45–47, 49–53, 55, 56), and one remained in a vegetative state (50).

### Results by type of neuromodulation technique

**Figure 2:**
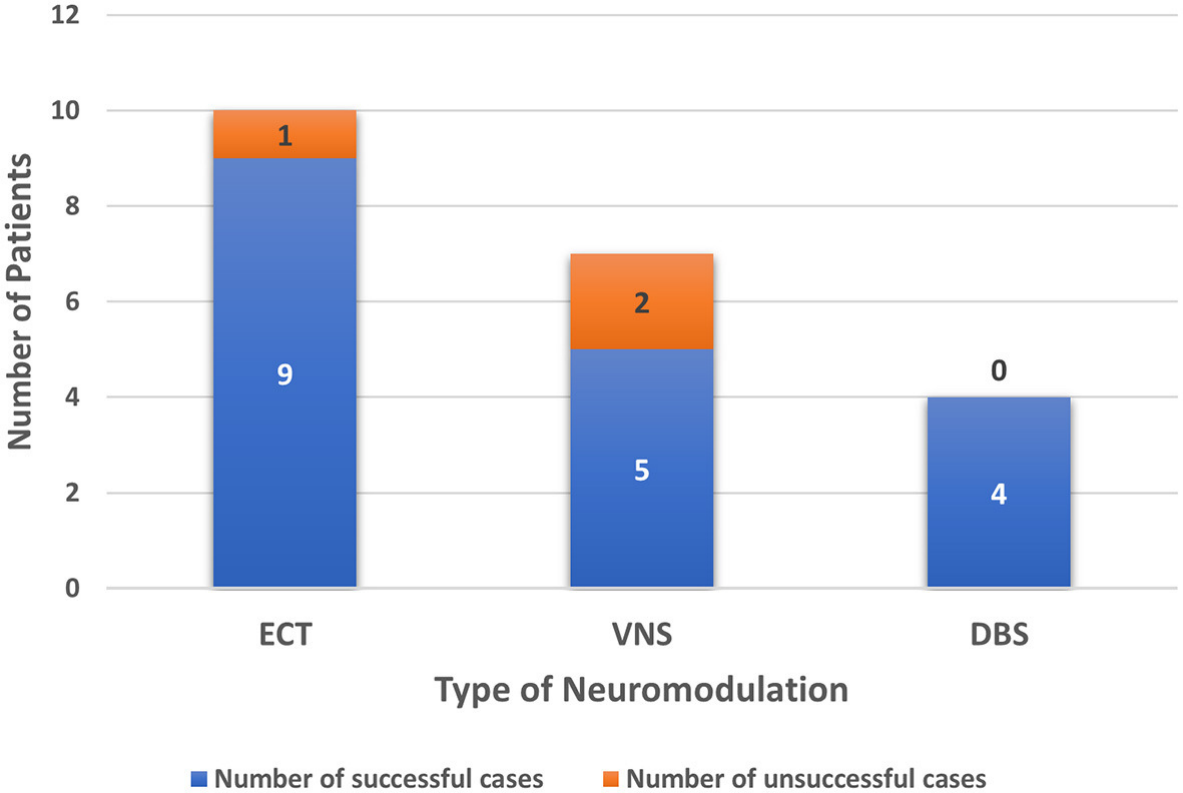
Number of patients reported for each type of neuromodulation including outcome.

**Figure 3:**
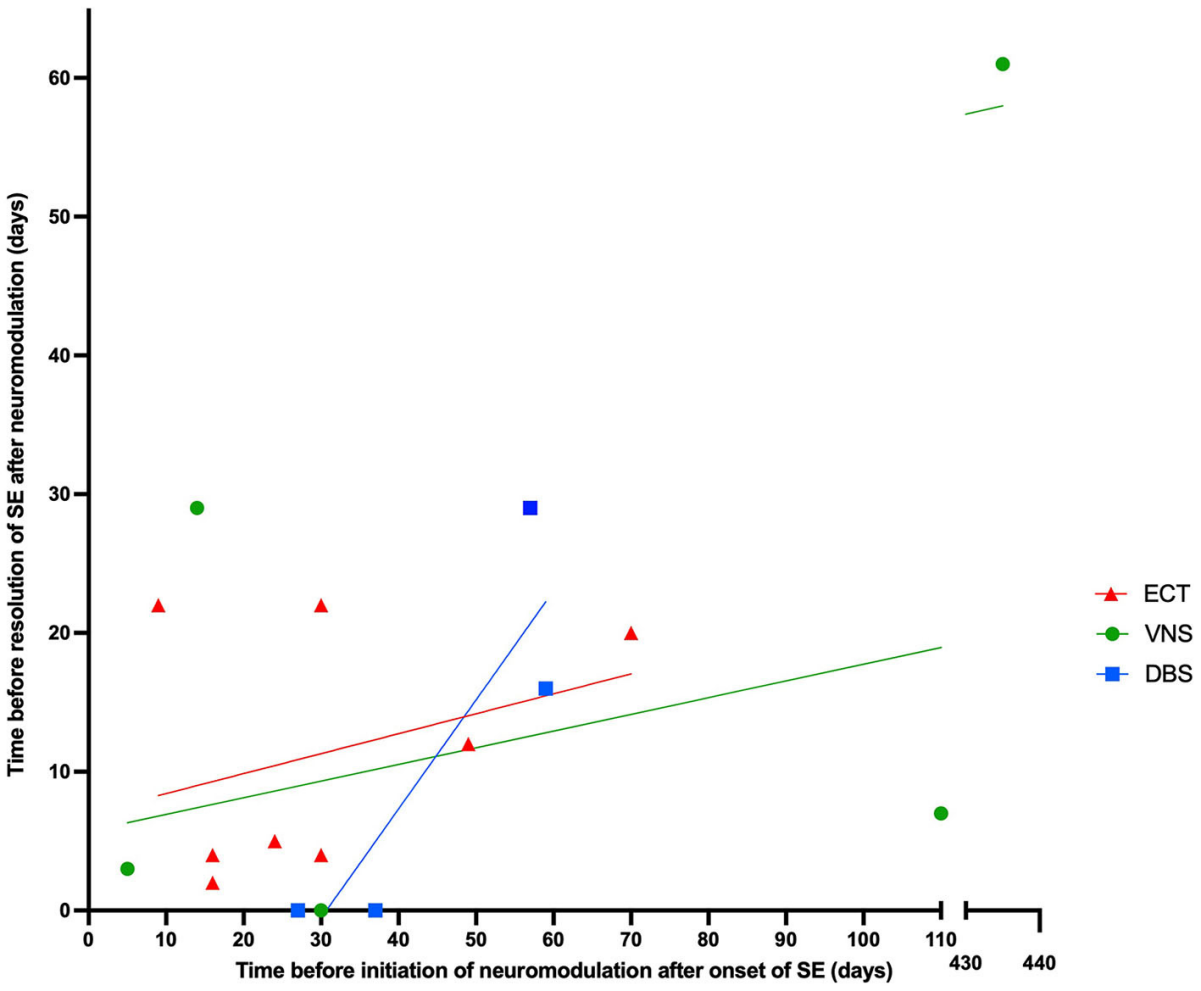
The relationship between time before initiating neuromodulation after onset of SE and time before resolution of SE after neuromodulation, categorized by type of neuromodulation technique.

ECT was performed in 10 patients and was successful in resolving SE in nine of those cases (44, 51–54, 56). VNS was implanted in seven patients and was successful in resolving SE in five of those cases (43, 46–49, 55, 57). DBS was implanted in four patients; all of them had implantation at the centromedian thalamic nucleus (CMN-DBS) with a 100% success rate (43, 45, 50) (Figure 2). Overall, neuromodulation techniques led to improvement in 18 out of 20 patients. VNS was discontinued for the patient from the Howell et al. (57) case study, who did not show any improvement and died of multiorgan failure. SE was never resolved for patient 2 from the Kamel et al. study (53), who also died due to multiple comorbidities, including multi-antibiotic resistant hospital-acquired pneumonia and acute renal failure. Figure 3 shows the time from the onset of SE until the initiation of neuromodulation and the period before the resolution of SE after starting treatment with neuromodulation. No neuromodulation technique could be suggested as superior to the others. Details about the case-by-case timeline for initiation of neuromodulation and the final outcomes are presented in Table 2.

**Table 2:** Characteristics of the patients, timelines for neuromodulation initiation, and outcomes.

**Authors/patient number**	**Type of NMD**	**Age**	**Gender**	**NORSE/FIRES**	**Etiology**	**Time from NORSE onset to initiation of NMD (days)**	**Time before resolution of SE after initiating NMD (days)**	**Outcome after NMD (D = days after onset of SE)**
Kurukumbi et al. (48)	VNS	25	Male	NORSE	Unknown	5	3	No SE or ES reported for 72 h after 3 days of VNS, succumbed to multiple comorbidities on D14
Alsaadi et al. (46)		46	Male	NORSE	Anti-NMDAR encephalitis	110	7	Weaned off midazolam after 1 week of VNS without any clinical or electrical seizures recurrences
Luo et al. (55)		3	Male	FIRES	Unknown	14	29	D43 seizure-free
Yamazoe et al. (47)		24	Male	FIRES	Anti-GluR autoimmune encephalitis	435^**^	61	Seizures completely disappeared after 2 months of VNS except for occasional eye deviation seizures
Howell et al. (57)/Pt. 7		14	Male	FIRES	Unknown	14	No improvement	No improvement over 15 days of VNS, died on D29 due to multiorgan failure
Espino et al. (49)/Pt. 1		37	Female	FIRES	Unknown	30	0	Cessation of SE 7 days after VNS implanted, but never seizure-free
Lehtimäki et al. (45)	DBS	17	Male	NORSE	CVID-associated encephalomyelitis	59	16	Resolution of SE and stepped down from neurointensive care on D75
Sa et al. (50)/Pt. 1		9	Male	FIRES	Unknown	27^*^	0	Almost abolishment of generalized seizures immediately after DBS implantation, seizure-free 33 days after (received anakinra 16 days after DBS)
Sa et al. (50)/Pt. 2		5	Male	FIRES	Unknown	37^*^	0	Almost abolishment of generalized seizures immediately after DBS implantation, which stopped completely 4 days later, remaining frequent focal seizures
Hect et al. (43)	VNS then DBS	11	Female	FIRES	Unknown	57	29	D85 onwards largely seizure-free, no abnormalities on serial EEG before discharge
Nath et al. (44)	ECT	3	Female	NORSE	Unknown	24	5	Seizure freedom lasted several hours to a day after each ECT treatment, persisted after fifth treatment, recurrence of 1–2 seizures a week later, resolved following two additional treatments except for some focal motor seizures
Kamel et al. (53)/Pt. 1		32	Female	NORSE	Unknown	30^*^	4^***^	SE resolved after 5 days (four ECT treatments)
Kamel et al. (53)/Pt. 2	ECT	41	Female	FIRES	Unknown	30^*^	No improvement	Seizures continued, died several days after initiating ECT due to multiple comorbidities
Kamel et al. (53)/Pt.3		26	Female	NORSE	Unknown	70^*^	20^***^	After fourth ECT treatment, seizure frequency decreased, SE resolved after another four treatments
García-López et al. (52)/Pt. 1		4	Male	FIRES	Unknown	60	Unknown	SE resolved after seven ECT treatments, kept having seizures
García-López et al. (52)/Pt. 2		32	Female	FIRES	Unknown	16	2	SE resolved after two ECT treatments, 2 months afterwards had auditory focal seizures without impairment of consciousness about every 10 days
García-López et al. (52)/Pt. 3		77	Female	NORSE	Unknown	16	4	SE resolved after four ECT treatments, living normal life without sequelae after discharge
Mirás Veiga et al. (56)		4	Male	FIRES	Unknown	49	12^***^	After 14 sessions, SE stopped, and EEG showed less frequent epileptiform activity
Tan et al. (54) Pt. 2		36	Male	FIRES	Unknown	9	22	Motor seizures resolved 2 weeks after eight ECT treatments
Chan et al. (51)		31	Male	FIRES	Unknown	30^*^	22	After first course of ECT, no sustainable improvement; second course given 8 days later; EEG stopped having ictal changes 10 days later

* Days after admission used, as onset of SE unknown.

** Corpus callosotomy performed after 14 months of SE (failed to terminate SE), VNS implanted 9 days after (~435 days since onset of SE).

*** Duration of successful ECT treatment was used, but SE could have been resolved earlier.

NMD, Neuromodulation; VNS, Vagus nerve stimulation; DBS, Deep brain stimulation; ECT, Electroconvulsive therapy; NORSE, New-onset refractory status epilepticus; FIRES, Febrile infection-related epilepsy syndrome; Anti-NMDAR, Anti-N-methyl-D-aspartate receptor; Anti-GluR, Anti-glutamic acid receptor; CVID, Common variable immunodeficiency; SE, Status epilepticus; ES, Electrographic seizures; EEG, Electroencephalogram.

### Medications administered

**Figure 4:**
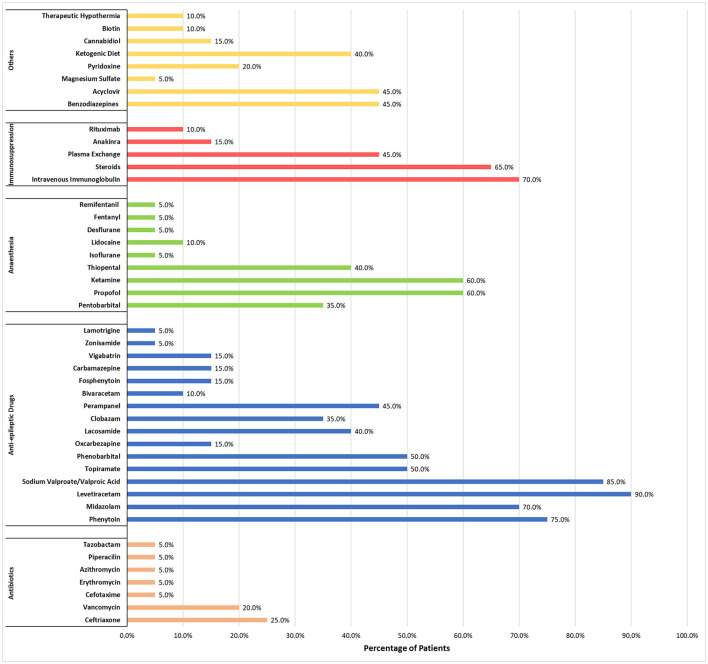
Percentage of patients who received each medication or treatment during management of NORSE/FIRES.

Both the mean and median number of drugs (including immune therapies and ketogenic diet) used in every patient was 14. Fourteen patients received immune therapies but only five patients received second-line immune therapies [three had anakinra (43, 49) and two had rituximab (50, 51)]. Figure 4 shows a complete list of other medications and treatments that were used, as well as the percentage of patients administered each, across the 15 studies included in this review. The most used anti-epileptic drugs were levetiracetam (90%) and sodium valproate/valproic acid (85%), while intravenous immunoglobulin (70%) and steroids (65%) were the most common for first-line immune therapies.

## Discussion

NORSE/FIRES represent a devastating condition with high mortality rates and poor neurocognitive outcomes (58). The necessity of rapid effective treatment is reflected in the timeline of the current therapeutic consensus (22). It is suggested that the poor outcome of NORSE, in both adults and children, is attributed to a combination of the duration of SE and the high rate of medical complications due to prolonged ICU stay and the high numbers and doses of anesthetics and antiseizure drugs required (19, 59, 60). It remains unclear whether this in fact is a consequence of the refractoriness of these cases, but on any occasion, the aim should be to reduce the iatrogenic burden. This could be supported by the adjunctive use of non-pharmacological techniques including neuromodulation.

The data collected in this review show that 18/20 NORSE/FIRES cases had a SE resolution after a trial of neuromodulation. Although the evidence is based on a small number of case reports/series with significant variability in time of application and techniques, neuromodulation techniques for these conditions appear to show potential benefit. Reasonably, the question arises regarding the proper time for consideration of a neuromodulation technique. The data in Figure 4 might suggest that SE resolution could happen earlier when the application of neuromodulation is performed closer to the date of NORSE onset, but the number of cases is very low and there are clear outliers. Based on these observations, we suggest that neuromodulation techniques, when available, could be considered earlier in the course of NORSE when the standard treatments have failed. Non-invasive neuromodulation techniques could be applied initially followed later by invasive neuromodulation techniques if SE continues. Even the possibility to use a different invasive technique if the first one was not associated with a good outcome has been reported. In one published case (43), unsuccessful VNS was followed by CMN-DBS with termination of SE.

Despite extensive diagnostic assessment, about 49.9% of the cases remain cryptogenic, creating difficulty in establishing a standardized treatment approach. Furthermore, it has been published that cryptogenic NORSE can be predicted by the use of a score with a sensitivity and specificity at 93.9% and 100% respectively without the need to wait for all the results of extensive antibody testing (61). In our review, 85% (*n* = 17) of the cases who had neuromodulation for NORSE/FIRES are cryptogenic, a percentage which is much higher than the described general cohorts of NORSE. This could be explained by the fact that neuromodulation is probably used later and as a last resort in cases where the diagnosis remains unclear and there is no benefit from standard treatments. As shown in this systematic review, 14/17 of the cryptogenic cases improved after neuromodulation, indicating that these techniques could start being considered as an add-on treatment option when the standard diagnostic testing returns without results and SRSE persists. Neuromodulation would not be expected to have interactions with the pharmacological treatments and thus it could be used as an add-on without necessarily waiting long for an established outcome of the other treatments, especially if the timepoint of 7 days has passed and initiation of second-line immune therapies have not provided benefit.

An immune-mediated inflammatory mechanism is considered responsible for many NORSE/FIRES cases and immune therapies are commonly used. According to the recent consensus, these should start within the first 72 h from SE onset and be followed by second-line immune therapies within the 1st week if SE has not been resolved (22). In a review of 161 patients with NORSE, 87.5% received immune therapy; however, the outcomes remained poor with mortality rates of 16.5% and 10.3% for NORSE and FIRES, respectively. A good functional outcome, when checking between immune therapies, was highest for treatment with glucocorticoids (40.4%) and second-line immune therapies showed less efficacy (rituximab, cyclophosphamide) which could be explained by the application to already refractory cases (62). In our review, immune therapy was administered in 16 out of 20 patients. A variety of immune therapies were used across different reports (a complete list can be found in Figure 4). As SRSE persisted, trials of neuromodulation were started, which were associated with good outcomes in 14 patients (43–47, 49–54, 56, 57). All four patients who did not receive immunotherapy also showed improvement after neuromodulation (48, 53, 55). Given the prolonged effect of immune therapies, it would be hard to conclude whether the positive outcomes were caused exclusively by neuromodulation but in three cases where neuromodulation (DBS) was stopped after the improvement, there was a recurrence of SE which was again resolved when neuromodulation was restored (45, 50).

The use of neuromodulation techniques in the management of RSE/SRSE remains inconsistent as we have previously described (63). This is also true for the literature data we present in this systematic review which suggests that three neuromodulation techniques (ECT, VNS, and DBS) have shown some encouraging results. The evidence is based on limited data, without a consensus for a common protocol of neuromodulation application. The variability is caused by different available techniques at each center, distinct expertise, and cost. Furthermore, NORSE is a clinical presentation, not a specific disease, and there is significant diversity regarding the causes. Importantly, the mechanisms by which neuromodulation affects SE are not elucidated.

ECT is a long-used treatment option for psychiatric disorders, with several theories for its mechanism of action. Although distinct from the other neuromodulation techniques since the applied stimulation is not chronic, studies in different neurological conditions have shown that ECT can have a neuromodulatory effect by modification of resting state functional connectivity and regional gray matter volume (64). Animal studies have shown that many biologic processes can be altered, causing changes in neuroendocrine function, levels of neurotransmitters, neuroplasticity, and epigenetics (65). Internalization of NMDA receptors has also been described in rats' hippocampus after ECT (66). VNS was introduced in 1988, has been tested in clinical trials, and, since then, it has been implanted in thousands of patients with drug-resistant epilepsy. Despite being used for more than 30 years, the mechanism of action is not entirely elucidated. There are suggestions that VNS influences the limbic structures' function by altering the concentration of GABA and glutamate (67). Norepinephrine and serotonin levels can also be influenced by VNS function through impact on the locus coeruleus and the dorsal raphe nuclei (68). Moreover, changes in the brain's functional connectivity have been proposed as another possible effect of VNS. Recent studies have shown that changes in synchronization in specific frequency bands are different between responders and non-responders (69). Alteration of functional connectivity was also seen in a study using data from stereo-EEG recordings in patients with VNS. The connectivity could be either increased or decreased but was found decreased in the only patient who was a VNS responder (70). Similarly, the way DBS exerts its effects remains obscure. A major difference from other neuromodulation techniques is that a specific brain region is directly stimulated. It is not clarified whether the therapeutic effect is caused by the stimulation of neurons, glial cells, or fibers (71) by inhibition mediated by activation of GABAergic afferents or the inactivation of voltage-gated currents (71, 72).

These suggested mechanisms possibly reflect a change in excitation/inhibition balance which might facilitate the early termination of SE. However, immunological changes have also been described as a result of neuromodulation. More specifically, the effects of anterior thalamic nucleus DBS on plasma pro-inflammatory cytokine IL-6 and the anti-inflammatory cytokine IL-10 on a population with drug-resistant epilepsy were explored recently (73). The authors found that the IL-6/IL-10 ratio decreased significantly over time following DBS treatment and responders had an increase in IL-10. In the same direction, there is evidence that VNS can have an impact on inflammatory disorders by evoking a protective decrease in pro-inflammatory cytokines and the pro- and anti-inflammatory cytokine balance can indicate a positive outcome of VNS (74, 75). As the available data about NORSE/FIRES grows, it appears that autoimmune encephalitis is the most common cause and cryptogenic NORSE cases are possibly immune-mediated, but unidentified autoantibodies or inadequate work-up cause a failure in cause establishment. Moreover, elevated pro-inflammatory cytokine/chemokine levels are found in many cases (76). The second-line immune therapies for NORSE interfere with inflammation-related interleukin action. Based on these observations, new studies exploring the possible anti-inflammatory effect and possible synergistic action of neuromodulation techniques would be of great interest and could possibly improve understanding of the delayed effect seen in a big number of cases.

This review has several limitations. The number of patients who have undergone neuromodulation for NORSE/FIRES is too low to provide robust results and allow guidance. Moreover, there is a high chance of significant reporting bias with successful neuromodulation cases being more likely to be submitted for publication compared to the ones where neuromodulation did not provide benefit. Furthermore, the grouping of cases under the umbrella of NORSE/FIRES might not be entirely accurate due to differences in diagnostic algorithms used in different centers and for some older cases. Similarly, the treatment approaches present major differences between patients, and this would be expected to have an impact on the published outcomes. Despite these drawbacks, we believe that this work provides meaningful data for neuromodulation treatment consideration in NORSE/FIRES.

## Conclusion

This systematic review attempts to present the available data on the use of neuromodulation for the treatment of NORSE/FIRES. Three neuromodulation techniques have been reported for NORSE/FIRES cases with encouraging outcomes, either with non-invasive (ECT) or with implantable devices (VNS and DBS). DBS caused the termination of SRSE in all four cases, but no neuromodulation technique appeared clearly superior to the others. The goal of neuromodulation remains the termination of SRSE as early as possible, aiming to reduce mortality; however, there is no evidence of differences in long-term outcomes. The application of neuromodulation has not been tested through randomized, prospective controlled clinical trials, as has most of the other available treatments for this devastating condition, but the existing data show some potential benefit of neuromodulation therapy, suggesting that these techniques could be considered within the course of NORSE.

## Data availability statement

The original contributions presented in the study are included in the article/Supplementary material, further inquiries can be directed to the corresponding author.

## Author contributions

IS, JK, and AV wrote the manuscript. JK and IS performed the literature review. JK performed the search and prepared the graphs. All authors reviewed and approved the final version of the manuscript.
