# Supplementary material for: Neuromodulation in new-onset refractory status epilepticus
Source: Front Neurol. 2023 Jun 14;14:1195844. doi: 10.3389/fneur.2023.1195844 (PMC10301751; doi:10.3389/fneur.2023.1195844)
Supplement: Supplementary file 1 [file Data_Sheet_1.docx]

**Appendix A: Search Terms on PubMed**

(Neuromodulation*[Title/Abstract] OR neuromodulatory*[Title/Abstract] OR neurostimulation*[Title/Abstract] OR DBS[Title/Abstract] OR VNS[Title/Abstract] OR "deep brain stimulation"[Title/Abstract] OR "vagus nerve stimulation"[Title/Abstract] OR "vagal nerve stimulation"[Title/Abstract] OR "vagus nerve stimulator"[Title/Abstract] OR "cortical stimulation"[Title/Abstract] OR "cranial nerve stimulation"[Title/Abstract] OR "responsive neurostimulation"[Title/Abstract] OR RNS[Title/Abstract] OR "closed-loop neuromodulation"[Title/Abstract] OR CLN[Title/Abstract] OR "closed-loop neurostimulation"[Title/Abstract] OR "open-loop neurostimulation"[Title/Abstract] OR "closed-loop VNS"[Title/Abstract] OR "closed loop neuromodulation"[Title/Abstract] OR "closed loop neurostimulation"[Title/Abstract] OR "open loop neurostimulation"[Title/Abstract] OR "closed loop VNS"[Title/Abstract] OR "closed-loop stimulation"[Title/Abstract] OR "closed loop stimulation"[Title/Abstract] OR "open-loop stimulation"[Title/Abstract] OR "open loop stimulation"[Title/Abstract] OR ECT[Title/Abstract] OR "electroconvulsive therapy"[Title/Abstract] OR tDCS[Title/Abstract] OR "transcranial direct current stimulation"[Title/Abstract] OR "transcranial direct-current stimulation"[Title/Abstract] OR TMS[Title/Abstract] OR "transcranial magnetic stimulation"[Title/Abstract])

**AND**

(NORSE[Title/Abstract] OR "new-onset refractory status epilepticus"[Title/Abstract] OR "new onset refractory status epilepticus"[Title/Abstract] OR "febrile infection related epilepsy syndrome"[Title/Abstract] OR "febrile infection-related epilepsy syndrome"[Title/Abstract] OR FIRES[Title/Abstract] OR SRSE[Title/Abstract] OR RSE[Title/Abstract] OR "super refractory status epilepticus"[Title/Abstract] OR "super-refractory status epilepticus"[Title/Abstract] OR "refractory status epilepticus"[Title/Abstract] OR "super-refractory SE"[Title/Abstract] OR "super refractory SE"[Title/Abstract] OR "encephalitis with refractory repetitive partial seizures"[Title/Abstract] OR AERRPS[Title/Abstract] OR DESC[Title/Abstract] OR PEE[Title/Abstract] OR "postencephalitic epilepsy"[Title/Abstract] OR "post-encephalitic epilepsy"[Title/Abstract] OR IHHES[Title/Abstract] OR "infantile hemiconvulsion-hemiplegia and epilepsy syndrome"[Title/Abstract] OR "febrile status epilepticus"[Title/Abstract])

**Filters:**Full text, Humans, English

**Date of search:** 09 February 2023
